# Pregnancy enhances facial recognition of anger: Transition from early to late pregnancy

**DOI:** 10.1192/j.eurpsy.2024.1686

**Published:** 2024-08-27

**Authors:** F. Minami, R. Iwata, H. Nishida, S. Kudo, M. Yamagishi, K. Kamiya, M. Mimura, J. Hirano, B. Yamagata

**Affiliations:** ^1^Neuropsychiatry, Keio University School of Medicine; ^2^ Warakukai Medical Corporation; ^3^Ebis Medical Clinic, Tokyo, Japan

## Abstract

**Introduction:**

Pregnancy and the postpartum period involve several physiological adaptations crucial for offspring care. Recent research has highlighted reproduction-related brain plasticity in human mothers. Associations with aspects of maternal caregiving suggest adaptive changes that facilitate a woman’s transition to motherhood. However, the dynamic changes that affect a woman’s brain are not merely adaptive, and they likely confer a vulnerability for the mental disorders. To elucidate the pathophysiology of psychiatric problems that occur during the perinatal period, gaining insights into the physiological changes in brain function due to pregnancy is crucial.

**Objectives:**

Although it has been hypothesized that pregnancy enhances social cognitive functions in mothers to adapt to the offspring care, there are few reports to support this hypothesis. This study aims to investigate whether social cognitive functions change during the first pregnancy, with a focus on maternal adaptation to offspring care.

**Methods:**

The study included a first pregnancy group and a never-pregnant control group. We conducted a prospective study comparing pregnant women between two-time points (T1, T2); at less than 21 weeks of gestation [T1] and those after 30 weeks of gestation [T2]. To assess the effects of pregnancy and gestational age (< 21 weeks or 30 weeks or more), both the control (never-pregnant) group and pregnant group were evaluated at two time points with similar intervals. The Emotion Recognition Task [ERT] of the Cambridge Neuropsychological Test Automated Battery (CANTAB) was performed to examine the emotion recognition of six basic emotions in facial expressions. We analyzed a cohort of 26 participants in the pregnant group and 25 in the control group. We performed a two-way repeated measures analysis of variance with pregnancy status and gestational period (T1, T2) as independent variables.

**Results:**

Significant interactions between group and time points (T1, T2) were observed only for Unbiased Hit Rate Anger (p<0.01); facial recognition accuracy for anger increased with the progression of pregnancy. There were no significant interactions for Unbiased Hit Rate Sadness, Happiness, Fear, Disgust, or Surprise.

**Conclusions:**

This is the first study to demonstrate that facial recognition of anger enhances with the progression of pregnancy, utilizing never-pregnant women as a never-pregnant control group. The results of this study contribute to the physiological effects of pregnancy on the brain and cognitive function and have potential for further study of perinatal mental health problems.

**Disclosure of Interest:**

None Declared

